# Building Professionalism Through Management Training: New England Public Health Training Center's Low-Cost, High-Impact Model

**DOI:** 10.1097/PHH.0000000000000693

**Published:** 2017-10-05

**Authors:** Kathleen MacVarish, Hope Kenefick, Anne Fidler, Bradley Cohen, Yuri Orellana, Karla Todd

**Affiliations:** New England Public Health Training Center at Boston University School of Public Health (Ms MacVarish, Drs Kenefick, and Fidler, and Ms Todd); and Boston Public Health Commission, Consortium for Professional Development (Messrs Cohen and Orellana).

**Keywords:** management, training, workforce, Public Health 3.0

## Abstract

**Context:**

Evolving practices, accreditation, and priorities established in Public Health 3.0 are adding to the long-identified need for management training among public health practitioners.

**Program:**

The New England Public Health Training Center is addressing this need with a flexible, open-source, 16-topic training program. The program is designed to build competencies for current and future managers, preparing them for their day-to-day tasks and for the kinds of adaptation suggested by Public Health 3.0 advocates.

**Implementation:**

The training program uses live expert instructors for 10 webinars and 2 in-person trainings. Experts have also created the content for multiple self-paced E-Learnings that trainees undertake in addition to the instructor-led sessions. A webinar platform with breakout rooms and an advanced learning management system allows for online discussion and mentor interaction. The course has now been offered, evaluated, and modified 3 times, and the materials are available for noncommercial use by the public health community.

**Evaluation:**

Using the Kirkpatrick training evaluation model, the recent cohort was satisfied (87.5%) with the training, reported identifying actions to apply information learned to their work (85.8%), and experienced statistically significant knowledge gains. Earlier trainees reported work-related behavior change.

**Discussion:**

Management training offers the hope of increasing professionalism; creating better, more effective workplaces and programs; and preparing practitioners for an evolving public health landscape. Early results indicate that NEPHTC's program, Managing Effectively in Today's Public Health Environment, is a useful tool in realizing that hope.

## Abstract 2

Supplemental Digital Content is Available in the Text.

## Context

A changing public health environment has led to a growing need for management training for public health professionals.^1^

The steady evolution of expectations and practices is changing the responsibilities of practitioners in ways that lead to new supervisory roles and the need for extensive coalition building, both beyond customary health department duties. The evolution has come in response to both societal and technological changes—changes often characterized as the arrival of Public Health 3.0,^2^ which recognizes use of data, partnership building, accreditation, and, ultimately, thriving communities as critical public health priorities. For example, large-scale data collection and analysis have opened new ways of identifying public health concerns and measuring outcomes. This, in turn, has brought to light issues of health inequity, expanding the scope of potential intervention and realigning missions. Local preparation for emergency response to attacks through mass violence, chemical, or biological means has grown—and received considerable funding—since the attacks of 2001. And training for public health professionals has expanded and improved with standards for professional competencies and continuing education.

The need for management training in the public health world had been recognized nationally for some time, recently summarized in a *Journal of Public Health Management and Practice* editorial, “Public Health Leadership and Management in the Era of Public Health 3.0,” which argued,

There is a need for a much more concerted, coordinated effort to build foundational, high-performance skills. For example, leadership development and management skills along with policy development, communicating persuasively, and systems thinking are necessary for effective coalition building, collective impact, and facilitation of strategic discussions within and across sectors.^1^

Regionally, that need had been reinforced by specific requests and suggestions for adding management training. Practitioners from sanitarians to public health nurses, health inspectors/agents, and health directors reported that their academic training did not include fundamental organizational management skills.

In 2012, the Boston University School of Public Health (BUSPH), which hosts the New England Public Health Training Center (part of the Public Health Learning Network created to support professional training in public health throughout the United States), set out to address the need for management training. The NEPHTC's goal was to create a curriculum and model that allow for a low-cost, time- and travel-efficient training program that other organizations are welcome to use, with the potential to make public health management training available nationwide. Two factors helped make this possible. First, the BUSPH team is supported by grants from both the federal Health Resources & Services Administration and the Massachusetts Department of Public Health. Multiple funding sources allowed the NEPHTC to invest in developing and implementing a management training program while still meeting other public health practitioner needs. Second, rapid improvement in online learning platforms made it possible to create a hybrid, in-person/online course, providing rewarding, effective training while minimizing time away from day-to-day responsibilities. The NEHPTC licensed this course under a Creative Commons Attribution-Non-Commercial 4.0 License, making it available for reuse and adaptation (http://www.nephtc.org/course/view.php?id=38).

## Approach

The BUSPH team set several larger goals for developing Managing Effectively in Today's Public Health Environment. The course needed to be adaptable to states and localities in New England and beyond, each of which might have local regulation, budgeting, and employment practices. The curriculum needed to provide value to trainees even if the presenting entity chose to truncate the course and not use the full set of topics and lessons due to budget or time limitations. The materials needed to be comprehensive with the expectation that many different expert instructors would be adapting and presenting them and would want to do so with limited time and limited expense to the presenting training entity. And the content needed to be applicable to the day-to-day tasks of practitioners and also help them adapt to the larger directions and concepts embodied in Public Health 3.0.

In January 2012, NEPHTC formed a curriculum planning team with members from the BUSPH, the Boston Public Health Commission, the Massachusetts Coalition for Local Public Health, the Massachusetts Department of Public Health, the Yale Public Health Training Center, and an independent management consultant. Initial planning consisted of 3 steps:

1. The committee contacted the UNC Management Academy for Public Health, commonly cited in the literature,^3^ which shared its course outline and slides, some of which were incorporated into the NEPHTC course outline, though the teaching methods and assignments of the 2 courses would diverge.
2. The team identified existing courses and curricula, including a core book, *Essentials of Public Health Management*.^4^
3. It reviewed competency goals from the 2010 Local Public Health Institute of Massachusetts Competency Report^5^ and the Council on Linkages Between Academia and Public Health Practice to ensure that the management course would help trainees develop competencies that met regional and national needs and expectations.

With those tools, the committee created a course outline for a 28-hour course run as a series of 2-hour weekly webinars. Team members from the BUSPH then refined the syllabus, outlining content for each of the topic areas and creating course assignments. In the process, the team changed its plan from an all-online class to a hybrid online and in-person format, replacing 2 of the webinars with 2 half-day classroom sessions to facilitate networking and personal interaction and content that would be less effective online. The second classroom session is the final day of the course and includes a graduation ceremony to celebrate student success. By May 2012, the framework of the course was set (Table 1).

**TABLE 1:** Management Topic Alignment with Public Health 3.0

Management Training Course Topic	Public Health 3.0 Key Components
From manager to leader	Leadership and workforce
Leading organizational change	
Recruiting and hiring	
On-boarding and coaching	
Labor law and collective bargaining	
Grievances and discipline	
Budgeting and resources 1	Flexible and sustainable funding
Budgeting and resources 2	
Grant writing	
Managing projects and teams	Data, analytics, and metrics
Program evaluation	
Quality improvement	
Community collaboration	Strategic partnerships
Getting started (orientation to public health)	Essential infrastructure
Setting the stage	
Marketing public health	

The BUSPH team members began recruiting subject matter experts to develop the individual lessons and lead the first course. The use of the webinars expanded the potential instructor talent pool and raised the quality of the instruction.

Over the following months, the experts created the lessons. By the end of 2012, the course was fully developed with assignments, activities, instructor slides, and detailed instructor notes. This original version had 14 sessions: 2 in-person classroom days and 12 two-hour interactive webinars held through the Adobe Connect webinar platform. The BUSPH team had also developed an evaluation and quality improvement plan for ongoing implementation.

In the first quarter of 2013, an independent committee of experts reviewed the course content and curriculum. Members included academics and practitioners from Boston Public Health Commission, BUSPH, Brown University, the University of North Carolina, the Local Public Health Institute, the Massachusetts Department of Public Health, the Massachusetts Association of Health Boards, and Yale University. By April 2013, the course was ready for marketing, with a launch in September.

The NEPHTC reached out to major regional public health associations, which helped alert members to the new course. The Local/State Advisory Committee, Massachusetts Health Officers Association, Massachusetts Environmental Health Association, and the Massachusetts Association of Public Health Nurses all participated in marketing. The Boston Public Health Commission's training office marketed internally to the state's largest municipal public health entity. Academic partners identified trainees from Connecticut and Rhode Island.

There were 33 enrollees for the initial 2013 cohort. Participants included new managers, including public health nurses, health inspectors, and a state environmental health inspector, along with more senior managers including city health department administrators, managers and directors, town public health directors, and state officials. Enrollees were not charged a fee for the course. Subsequent cohorts were charged minor fees for incidental expenses. The completion rate for the first cohort was 82%.

The course was evaluated and modified, then offered beginning in May 2015, and again beginning November 2016 in Massachusetts. The second and third cohorts had 28 and 53 enrollees and an 89% and 94% completion rate, respectively. To date, in Massachusetts 102 trainees have completed the course. An adaptation of the course was offered in September 2016 in Maine, which 17 public health professionals completed. A comparison of the Maine versus Massachusetts offering of the course, titled “basic” and “expanded,” requiring different levels of staff, experts, and learning technology resources, can be found at Supplemental Digital Content Table A, available at http://links.lww.com/JPHMP/A394.

Based on feedback, observation, and evaluation of the classes, the management training course has been altered and improved each time it has been offered since the 2013 class. In 2015, NEPHTC added group mentors, 1 per 5 or 6 students, to improve engagement and moderate discussions. The mentors are current or retired health department leaders identified as effective group leaders. In 2016, several webinar sessions were converted to E-Learnings that are available to the entire public health community, even for those not enrolled in the course. New E-Learnings included onboarding new employees, coaching employees, grant writing basics, and marketing public health.

Also important was hosting the course on an online learning platform. In 2016, the NEPHTC identified the online learning management system Moodle (https://moodle.org/) as offering a better experience and features more suited to the goals and learning strategies of the management training course. While Moodle is an open source platform, use requires support of educational technology and instructional design staff.

The goal of using Moodle was to increase learner-to-learner interaction and to improve ease of use by instructors, learners, and mentors. The system made discussion groups easy to access and moderate. Before using Moodle, discussion and assignments went back and forth through e-mail, which was hard to follow and easy for learners to lose track of. Moodle put all of the course materials, schedule, assignments, and conversations in a single online classroom.

### Course adaptation

Managing Effectively in Today's Public Health Environment was designed to be customized, truncated, or expanded. The Boston Public Health Commission chose the latter—expanding the general management curriculum with extended time for internal discussions and content developed by its internal training team. The additional content targeted health equity, racial justice, community engagement, and application of course content to internal practices and approaches. It offered the additional benefit of allowing trainees to have discussions with senior administrators within BPHC. The BPHC trainees made up roughly half of the pilot class and a large proportion of the 2016-2017 class.

In Maine, the local NEPHTC training site, the University of New England, ran the management program with fewer training topics and without the online Learning Management system. The NEPHTC worked with Maine public health directors and content experts to adapt, modify, and develop a curriculum suited for Maine professionals. Starting with the 16 topics from BU, they created a 6-session course delivered in 3 daylong in-person classroom training sessions and three 1-hour to 1.5-hour interactive webinars. With fewer experts and less management required, the Maine course fits the needs of area practitioners while remaining within local limitations in budget and staffing. Trainees came from the Maine Centers for Disease Control and Prevention and health departments in Portland and Bangor.

Several months of faculty and staff time, and consulting fees for experts, were incurred for developing the curriculum and delivery processes in year 1. This work is freely available now, so implementation costs will vary depending on the topics chosen and delivery modality. For a participating organization, or an organization choosing to use the curriculum, in whole or in part, with internal experts, costs would be driven by staff time reviewing the curriculum, planning the in-person or webinar sessions, meeting expenses, and chosen method of evaluation. Beyond staff time and payment for a bus to the second live class and graduation, the Boston Public Health Commission had no out-of-pocket costs for its adaptation and delivery of the program. An organization wanting to adapt and deliver some of the trainings could potentially do so with no out-of-pocket expenses, incurring cost only in staff time. An organization wanting to run a full-length management program with a learning management system, mentoring and hired experts, would need both staff time and out-of-pocket expenses.

## Evaluation Strategy

Each session of Managing Effectively in Today's Public Health Environment was evaluated by NEPHTC's evaluator on the basis of the available data. Since its inception, the NEPHTC has evaluated the course at levels 1 and 2 of the Kirkpatrick Model of training evaluation^6^ using a Likert Scale^7^ to assess agreement with a series of statements about the course and by administering a quiz before and after training (summary shown in tables). In 2015, with the second cohort, the evaluation expanded to include interviews with a subset of course graduates 7 months after course completion to understand the impact of training on their job performance (ie, Kirkpatrick level 3).^8^ The interviews were part of a pilot effort to understand the impact of 4 NEPHTC trainings on trainees' work. A summary table of the 4 levels of Kirkpatrick Model of Training Evaluation and evaluation used for this management course can be found at Supplemental Digital Content Table B, available at http://links.lww.com/JPHMP/A395. Interviews are also planned to assess level 3 impact with the 2017 cohort. The evaluation plan informed quality improvement of content, modalities, instructors, and mentors (not shown). All evaluation materials are shared with the public health training community (http://www.nephtc.org/course/view.php?id=38).

Overall findings from 3 implementations of the management course suggest that students were generally satisfied with the course sessions, pre- and postwork, instructors, and mentors. Course mentors believe that they had a positive influence on students' experiences in the course and were satisfied with their experience as a mentor. The number of trainees who expected to apply the training to a state or national certification ranged from roughly 40% in 2013 to 17% in 2017.

### Level I: Reaction

Reaction to the course, content, staff, and overall value has been positive. The 2017 evaluation included topic-by-topic feedback. Across all sessions in the 2017 cohort, an average of 87.5% of participants were satisfied, 90.5% felt that the information was presented clearly, 89.7% felt that their understanding of the subject matter improved, and 85.8% identified ways to apply what they learned to their work (Table 2).

**TABLE 2:** 2017 Trainee Agreement (Agree or Strongly Agree) With Statements About the Training Sessions

Course Topic	The Information Was Presented in Way I Could Clearly Understand			I Was Satisfied With This Training Overall		
	N	n	%	N	n	%
Introduction	39	38	97.4	38	36	94.7
Organizational change	49	49	100.0	49	49	100.0
Labor laws	54	51	94.4	54	52	96.3
Recruiting	44	43	97.7	44	40	90.9
Grievances and discipline	44	37	84.1	46	38	82.6
Budgets and resources	37	36	97.3	35	32	91.4
Budgets and resources	44	39	88.6	45	39	86.7
Project management	40	37	92.5	40	35	87.5
Quality improvement	40	31	77.5	39	33	84.6
Program evaluation	41	35	85.4	43	32	74.4
Community collaboration	39	28	71.8	42	26	61.9
Leadership	47	45	95.7	46	44	95.7
Average	43	39	90.2	43	38	87.2

In addition, 75% or more of 2017 course participants agreed or strongly agreed with statements (ie, about course assignments, techniques for engaging learners, use of a team approach) that suggest the participants felt positively about the overall approach and implementation of the course.

### Level II: Learning

The mean quiz scores were higher at posttest than at pretest for all 3 cohorts. For the 2017 cohort, a paired samples *t* test indicates that the improvements in scores seen from pretest (*M* = 49.5, SD = 12.6) to posttest (*M* = 72.9, SD = 16.2) are the result of the training and the training was effective at improving trainee knowledge, *t*(48) = −9.140, *P* < .005 (Table 3).

**TABLE 3:** Pre- and Posttest Quiz Data

Course Year	Average Score and Range Precourse	Average Score and Range Postcourse
2017 (%)	Average: 50	Average: 73
	Range: 30-86	Range: 29-100
2015 (%)	Average: 42	Average: 69
	Range: 14-64	Range: 36-93
2013 (%)	Average: 62	Average: 84
	Range: 47-93	Range: 60-100

Trainees also self-report knowledge gain and likely application to workplace (Table 4).

**TABLE 4:** 2017 Trainee Agreement (Agree or Strongly Agree) With Statements About the Training Sessions

Course Topic	My Understanding of Subject Matter Improved as a Result of Having Participated in This Training			I Have Identified Actions I Will Take to Apply Information I Learned From This Training to My Work		
	N	n	%	N	n	%
Introduction	39	37	94.9	39	36	92.3
Organizational change	49	49	100.0	49	49	100.0
Labor laws	54	53	98.1	53	50	94.3
Recruiting	44	40	90.9	43	38	88.4
Grievances and discipline	46	40	87.0	45	38	84.4
Budgets and resources	37	33	89.2	36	32	88.9
Budgets and resources	46	45	97.8	44	34	77.3
Project management	40	34	85.0	40	36	90.0
Quality improvement	41	34	82.9	42	33	78.6
Program evaluation	43	33	76.7	43	31	72.1
Community collaboration	42	31	73.8	40	26	65.0
Leadership	46	44	95.7	43	41	95.3
Average	44	39	89.3	43	37	85.6

### Level III: Behavior

Because little is known about how best to conduct level 3 evaluation of training, the NEPHTC conducted a pilot project involving brief (15-20 minutes) telephone interviews with 15 graduates of 4 NEPHTC courses to understand how graduates perceive the trainings in which they participated affected their work and to advise the NEPHTC on methods (ie, interviews vs surveys) for gathering level 3 data in future. A total of 31 interview participants were selected at random from lists of course graduates. Of the 31 contacted, 15 (48.4% response rate) completed interviews. Three participants completed and, therefore, commented on 2 of the trainings involved in the interview project. Six of those contacted were not responsive, 4 had inactive e-mails, 1 was on leave from her work, and 2 declined because they were not in a position to use the knowledge and skills learned in the training due to retirement and a change in job responsibilities. An interview guide with open-ended questions was developed for the project based on a deductive approach that assumed the training had an impact on trainee job performance and sought trainee reflection, observation, and confirmation of how the training influenced their work. Detailed notes were taken during the calls for the purpose of identifying common and divergent themes and to capture direct quotations. The ways in which training affected trainees' work across the 4 trainings differed by the content covered in each training. Although the project involved only a small subset of management course participants (n = 5), these graduates provided consistent and positive comments and all identified ways in which the training had a positive influence on their work. The interview participants attributed improvements in their job performance to training in the following areas:

- Managing difficult employees
- Hiring
- On-boarding new staff
- Developing and supporting staff
- Forming and coordinating effective teams
- Budgeting
- Advocating for resources

The average time these practitioners had been employed in public health was 19 years, with a range of 10 to 27 years. Despite having been managers for many years, all felt that they learned something important from the course. One explained that, “In public health, folks ascend into management roles often with little training in how to manage.”

They attributed several changes in how they do their work to the training, including the hiring, on-boarding, and supporting of staff. One noted that he hired 2 people shortly after the course concluded and that he applied what he had learned in the training, particularly the information about what not to ask in an interview. Thereafter, he prepared packets with resources and checklists that would help the new staff orient to their jobs. He checks in with staff regularly to see how they are doing and offers support. He offered: “Before, my mentality [with new staff] was sink or swim. Now I feel better prepared to support their professional development.”

Another trainee felt that the most valuable part of the course for him was the information he learned about conflict resolution and working effectively with unions. He supervises unionized employees and feels much clearer about proper documentation procedures should he need to take disciplinary action in the future.

One respondent felt that she gained skills for managing her team better, helping them to work together more effectively, and how to deal with difficult personalities in the workplace. She also described improvements in her ability to address conflict in the workplace and described herself as a better listener as a result of the course. Another described the skills he gained related to staff management and conflict:

The course definitely influenced my practice, particularly how I handle difficult employees and conflicting personalities among staff, as well as how I motivate staff and build consensus. Before the course, I never really thought about these things. Now, I am more strategic about addressing problems and get to solutions more readily.

One described how the course influenced both the way he supports his team and how he works with others in his department.

I feel like I am a stronger advocate for my team and do a better job of arguing for what they need to do their jobs. I also do a better job of connecting with other areas within the department now and feel more connected to the big picture of what is going on.

The interview participants also described the information on budgeting as helpful to them. One, a longtime manager, said that all of the budgeting information reviewed in the course was new to her. Another applied the information he learned about evaluation and advocating for resources during the last budget season. He feels that using data allowed him to make more compelling arguments for additional resources. He added: “I have also integrated evaluation into my work now, doing continuous improvement and looking at everything through a lens of how best to use the limited resources I have to do work more effectively.”

The trainees particularly appreciate the resource materials provided and have referred to them since completing the course.

## Discussion

Management training is often overlooked in many fields in which practitioners become managers, but it can make a substantial difference in operational efficiency, staff satisfaction and effectiveness, and in quality improvement.^9^,^10^ In the larger context, management training can help prepare public health practitioners to address the kinds of imperatives brought to the fore in Public Health 3.0, including better use of data, a wider net for funding, and close collaboration with outside entities in order to undertake “cross-sectoral environmental, policy- and systems-level actions that directly affect the social determinants of health.”^2^^(p6)^ This program is intended to instill openness and competency for change, one of the biggest obstacles to evolving public health priorities.

In developing, delivering, and marketing Managing Effectively in Today's Public Health Environment, New England Public Health Training Center met key needs of public health practitioners while staying within its larger mission: developing practical, relevant trainings that are low cost to the trainee and minimize time away from work for health department employees.

Across all sessions in the 2017 cohort, an average of 87.5% of participants were satisfied, 90.5% felt the information was presented clearly, 89.7% felt their understanding of the subject matter improved, and 85.8% identified ways to apply what they learned to their work. Average posttest scores were higher than average pretest scores for all 3 cohorts, and a paired samples *t* test for the 2017 cohort indicates that the improvements in scores seen from pretest (*M* = 49.5, SD = 12.6) to posttest (*M* = 72.9, SD = 16.2) are the result of the training, *t*(48) = −9.140, *P* < .005.

Individual pre- and posttest scores were not available for analysis by the evaluator for the 2013 and 2015 cohorts. A pilot effort to understand the Kirkpatrick level 3 impact of 4 NEPHTC trainings offered preliminary data to suggest that the management course influenced the practice of a subset of 2015 graduates and aided in planning future level 3 evaluation efforts. Although similar findings were found across the 5 interviews, a larger sample would have been desirable. However, budget and timeline limited the ability of the evaluator to pursue additional interviews for the pilot project. A larger data collection effort devoted to understanding the level 3 impact of the management course is planned for summer 2017 and will be informed by the results of the pilot interviews.

Although the evaluation process is evolving, the 3 levels of evaluation provided findings that demonstrate the value to trainees, the utility of the approach, and the quality of the implementation. Through the use of a Creative Commons Attribution-Non-Commercial 4.0 License and further marketing, the content can be widely shared and adapted, making it a resource for the entire public health community.

## Strengths and Limitations

Enrollment in the NEPHTC management training course has demonstrated the significant demand in the public health community for this kind of training. The trainees have been engaged and have reported benefiting from the course. The NEPHTC's significant investment in the course design, platform, and instruction has resulted in a low-cost program that attracts enrollment. The hybrid, webinar/in-person/E-Learning format allows for high-caliber instruction by recognized experts, flexibility for trainees to work and communicate asynchronously, and the opportunity to develop camaraderie with classmates and mentors. Finally, the course can help meet accreditation requirements (Domain 8: Workforce and Domain 11: Administration and Management^11^) and introduces many Public Health 3.0 concepts in a practical, real-world context.

Covering a large number of topics, however, means limiting the depth of the content. Each of the topic areas could be its own course. While trainees report better handling of their management duties, they have really begun to study only management and leadership skills. Similarly, there has been a wide range of experience and seniority among the trainees. Some trainees found aspects of the course too basic; others thought some of the content was too advanced.

**Implications for Policy & Practice**

- An up-front investment in developing a training program with a fully developed curriculum and complete instructor guidance can lead to low-cost, adaptable, open-source implementation, potentially reaching a broad swath of practitioners.
- While vertical, specialty training has been the focus of public health continuing education, management training is a core need that cuts across job titles and seniority levels.
- Management training represents a significant way to improve the likelihood of continued success in a rapidly changing public health environment, both for individuals and for their programs and practices.

## Supplementary Material

SUPPLEMENTARY MATERIAL
