# Supplementary material for: Building Professionalism Through Management Training: New England Public Health Training Center's Low-Cost, High-Impact Model
Source: J Public Health Manag Pract. 2017 Oct 5;24(5):479–86. doi: 10.1097/PHH.0000000000000693 (PMC6078487; doi:10.1097/PHH.0000000000000693)
Supplement: SUPPLEMENTARY MATERIAL [file jpump-24-479-s001.docx]

TABLES FOR PRACTICE FULL REPORT

BUILDING PROFESSIONALISM THOUGH MANAGEMENT TRAINING: NEPHTC’S LOW-COST, HIGH-IMPACT MODEL

SUPPLEMENTAL TABLE A.

|  | Expanded Program | Basic Program |
| --- | --- | --- |
| State | Massachusetts | Maine |
| Cohort (number completing) | 50 | 17 |
| Topics | 15 | 6 |
| Training Staff | 1 course manager  12 experts  8 mentors  1 teaching assistant  1 technologist | 1 course manager  3 experts  1 teaching assistant |
| Live meetings | 2 | 3 |
| Webinars | 10 | 3 |
| Self-Paced Trainings | 5 | 0 |
| Communication | Learning Management System | Email |
| Time Span | 18 weeks | 10 weeks |
