# Supplementary material for: Building Professionalism Through Management Training: New England Public Health Training Center's Low-Cost, High-Impact Model
Source: J Public Health Manag Pract. 2017 Oct 5;24(5):479–86. doi: 10.1097/PHH.0000000000000693 (PMC6078487; doi:10.1097/PHH.0000000000000693)
Supplement: SUPPLEMENTARY MATERIAL [file jpump-24-479-s002.docx]

TABLES FOR PRACTICE FULL REPORT

BUILDING PROFESSIONALISM THOUGH MANAGEMENT TRAINING: NEPHTC’S LOW-COST, HIGH-IMPACT MODEL

SUPPLEMENTAL TABLE B.

| Kirkpatrick Model of Training Evaluation | | | Evaluation methodology |
| --- | --- | --- | --- |
| Level 1 | Reaction | Training is favorable, engaging and relevant to jobs | Likert scale for pre- and post-course self-evaluation |
| Level 2 | Learning | Trainees acquire intended knowledge, skills, attitude, confidence, and commitment | Pre- and post-course quiz |
| Level 3 | Behavior | Trainees apply what they learned when back on the job | Open-ended interview questions; answers analyzed using qualitative thematic analysis |
| Level 4 | Results | Targeted outcomes or impact occur as result of the training | N/A |
